# Prevalence and determinants of unmet needs for hypertension care among the older population in Selangor: cross-sectional study

**DOI:** 10.1186/s12961-022-00915-1

**Published:** 2022-11-29

**Authors:** Shamsul Azhar Shah, Megumi Rosenberg, Dzulfitree Ahmad, Saharudin Ahmad, Nazarudin Safian, Yugo Shobugawa

**Affiliations:** 1 Department of Community Health, Faculty of Medicine, Universiti Kebangsaan Malaysia, Cheras, 56000 Kuala Lumpur, Malaysia; 2 Centre for Health Development, World Health Organization, Kobe, Japan; 3 Department of Family Medicine, Faculty of Medicine, Universiti Kebangsaan Malaysia, Kuala Lumpur, Malaysia; 4 Department of Active Ageing, Niigata University Graduate School of Medical and Dental Sciences, Niigata, Japan

**Keywords:** Hypertension, Unmet needs, Ageing, Older people, Care cascade

## Abstract

**Background:**

Hypertension is a major cause of morbidity and mortality worldwide. Although multiple studies have assessed the prevalence of hypertension among older people, no study has examined the unmet need for hypertension care among older people in Malaysia. This study uses the hypertension care cascade to identify the prevalence of unmet needs for hypertension care and their determinants among the older population in Selangor.

**Methods:**

This is a cross-sectional study involving a total of 1204 participants recruited from different areas in Selangor. A face-to-face interview was conducted using the Bahasa Malaysia version of the Japan Gerontological Evaluation Study questionnaire. The inclusion criteria were Malaysians aged ≥ 60 years who could converse in Bahasa Malaysia.

**Results:**

Among the 637 participants with hypertension, 18% (117) had not been previously screened but were found to have BP ≥ 140/90 mmHg, 21% (136) were undiagnosed, 3% (17) were untreated, 42% (267) were treated with antihypertensive medication but still had high blood pressure, and 16% (100) had hypertension that was controlled with medication. The hypertension care cascade demonstrates that 18% (117) of those with hypertension had never been screened for hypertension; 26% (136/520) of those who were screened never received a diagnosis; 4% (17/384) of those who were diagnosed did not receive treatment; and 73% (267/367) of those who were treated did not reach the threshold for control. The prevalence of total unmet needs was 84% (537/637). Statistically significant determinants of having any unmet need for hypertension care were smoking status and medical history, with adjusted odds ratios and 95% confidence intervals (CIs) in the multivariate analysis of 0.5 (95% CI: 0.3–0.9) for being a smoker, 2.8 (95% CI: 1.1–6.9) for having a history of stroke and 1.6 (95% CI: 1.0–2.5) for having a history of diabetes mellitus.

**Conclusions:**

The prevalence of unmet need for hypertension care among the older population in Selangor is 84% (537/637), which is alarmingly high. This study highlights where and how much of the loss of care for hypertension happens in the care cascade and provides insight into the efforts required to improve effective service coverage to manage the increasing burden of hypertension associated with population ageing.

## Background

Hypertension is the most prevalent vascular diseases globally. It is considered one of the main risk factors for the cardiovascular, cerebrovascular and peripheral vascular diseases that include coronary disease, stroke, peripheral artery disease, renal disease and heart failure [1]. It is estimated that 1.13 billion people have been diagnosed with hypertension worldwide and that the disease affects 1 in 4 women and 1 in 5 men. Two thirds of the hypertensive population live in low- and middle-income countries. Among those with hypertension, only 20% have well-controlled blood pressure [2]. The older population is the most rapidly growing subgroup globally, and more than 90% of individuals who are normotensive at age 55 will develop hypertension during their remaining life span [3]. According to the Seventh Report of the Joint National Committee on Prevention, Detection, Evaluation, and Treatment of High Blood Pressure, hypertension occurs in more than two thirds of individuals after the age of 65 years [4].

In Malaysia, cardiovascular disease has been one of the leading causes of death for the past 40 years. The relationship between blood pressure and the risk of cardiovascular disease events is continuous, consistent and independent of other risk factors. Although the prevalence of hypertension is high in Malaysia, unfortunately, levels of awareness, treatment and control are low, thus creating a high burden of disease [5]. A study in Malaysia showed a rising trend in the prevalence of hypertension among adults aged ≥ 30 years, from 32.9% in 1996 to 43.5% in 2011 [6]. About 2 out of 5 older people in Malaysia (aged ≥ 60 years) have been diagnosed with hypertension, with the highest prevalence (65%) among those aged ≥ 70. In 2019, the overall prevalence of known hypertension among adults aged ≥ 18 years in the country’s most populous state of Selangor was 12.7%, as reported in the National Health and Morbidity Survey [7].

Despite Malaysia having an easily accessible and highly subsidized public health system, significant barriers to effective hypertension management still exist [8]. One promising approach to assessing these is to use care cascades to identify gaps or loss of care across the continuum of chronic disease care, from screening to diagnosis, treatment, and control. Yan et al. define unmet needs as the total loss across all care cascade levels [9]. An unmet need is an indicator that is widely used to evaluate the service capability of a health system as it relates to health outcomes, financial risk protection, improvements in efficiency, and responsiveness to individuals’ reasonable health expectations [10].

Currently, no study offers insight specifically into the prevalence of the unmet need for care among older (> 60 years old) hypertensive patients in Malaysia. It is crucial to identify the extent of population coverage of screening, diagnosis, treatment, and control through the use of the hypertension care cascade to help inform policy-makers and assess the need for health system interventions. Thus, this study aims to identify the prevalence of unmet needs for hypertension care among the older population in Selangor and the risk factors for loss of care.

## Methods

### Study design

A cross-sectional study was conducted in four areas in Selangor, Malaysia, among the population aged ≥ 60 years, from 1 December 2018 to 30 April 2020. The state of Selangor was chosen for the study because it is the most populous state in the country, with 6.53 million inhabitants in 2020. It reflects the diversity of Malaysia in terms of people and living conditions, and it includes all major ethnic groups, namely Malaysian, Chinese and Indian.

The sampling method used in this study has been reported previously by Shah et al. [12] and Safian et al. [13]. Official public data about district administrative units and their population were used as the sampling frame. In Selangor, there are 9 districts and 177 subdistricts. Hulu Langat, with a population of more than 1 million, was chosen as representative of urban regions, while Kuala Selangor, with a population of 0.2 million, was chosen to represent rural areas. Multistage cluster sampling was performed to reflect sampling probability proportionate to the size of the older population. The primary sampling units are districts, specifically Hulu Langat and Kuala Selangor, and the secondary sampling units are subdistricts. Sixteen subdistricts were chosen: seven from Hulu Langat and nine from Kuala Selangor. Ten towns or villages were randomly selected from each subdistrict for the third sampling unit. In a typical subdistrict, there are approximately 30–50 towns or villages. The household registries for the selected areas were obtained with permission from the appropriate village head and used as the sampling frame for households and individuals. A random sampling of households with an older individual was carried out.

The Kish grid was used to select the participant when more than one older adult in a chosen household was eligible for the analysis. The sample size was calculated using the equation *n* = *Z*^2^[(*P*(1 − *P*)/*e*^2^)], where *Z* is the confidence level, *P* is the prevalence of good health among older people and *e* is the error margin. Using *Z* = 1.96, *P* = 0.3 (an indicative estimate based on a previous analysis of older individuals in Japan used in the absence of corresponding data for Malaysia) and *e* = 0.05, the initial sample size was 322. The design effect of 1.5 and the two groups of estimates (urban and rural) needed for the survey results were multiplied by this initial sample size, arriving at 966 participants. To account for an estimated 20% nonresponse rate, 966 was divided by 0.80, resulting in a total target sample size of 1207. We successfully recruited 1204 respondents, with a response rate of 99.8%.

This study uses interviewer-assisted questionnaires. Before the interview, respondents were briefed using detailed study descriptions with information sheets and consent forms. After the respondents signed the consent document, trained research assistants conducted face-to-face interviews in a private environment, which lasted 40–50 minutes. A culturally and linguistically adapted Bahasa Malaysia version of the questionnaire from the Japan Gerontological Evaluation Study was used [14], which incorporates multidimensional variables for assessing healthy ageing. The inclusion criteria for respondents were (1) age at least 60 years and able to converse in Bahasa Malaysia, (2) registered as a resident of Malaysia (household registries were used for the sampling frame), (3) living at home and (4) able to understand and give informed consent to participate in the study. However, individuals were excluded if they were uncooperative or had a score of less than 7 on the Abbreviated Mental Test [15] during the screening questions because this indicated the possibility of dementia. Other exclusion criteria were living in a nursing or care home.

### Covariates

The covariates analysed in this study were divided into four groups: (1) sociodemographic, such as age group, the residence area of the respondent (rural or urban), gender, ethnicity and marital status; (2) health and functional statuses, such as body mass index (BMI) and activities of daily living (ADL); (3) high-risk behaviours for hypertension, such as smoking and drinking alcohol; and (4) medical history, including diseases such as stroke, cardiovascular disease, diabetes and dyslipidaemia.

Age groups were categorized as 60–64, 65–69, 70–74, 75–79, 80–84, 85–89 and 90–94 years. Gender was classified as male or female. Marital status was categorized as married or other, which included being single, divorced or widowed. Locality was divided into rural or urban. Ethnic groups were categorized as Malaysian, Chinese or Indian, and other for those who did not belong to the major ethnic groups mentioned above. BMI classification was based on measurements of weight and height, which were each measured twice during the interview. Using the Malaysian BMI classification for reference, underweight was defined as BMI < 18.5 kg/m^2^, normal weight as BMI = 18.5–22.9 kg/m^2^, overweight as BMI ≥ 23 kg/m^2^, pre-obese as BMI = 23.0–27.4 kg/m^2^, obesity class I as BMI = 27.5–34.9 kg/m^2^, obesity class II as BMI = 35.0–39.9 kg/m^2^ and obesity class III as BMI ≥ 40 kg/m^2^ [16].

Using the criteria in the 2018 edition of the Malaysian clinical guidelines on managing hypertension, in this study hypertension was considered to be present if (1) a participant had an average systolic pressure ≥ 140 mmHg or an average diastolic pressure ≥ 90 mmHg, or both; or (2) the participant reported being diagnosed with hypertension previously; or (3) the participant reported being treated with antihypertensive medication in the past 2 weeks [17]. Blood pressure measurements were taken twice at least 5 minutes apart, with the right arm supported at heart level, using an automatic digital blood pressure monitor. Participants in the study were advised to abstain from smoking, exercising or eating during the 30 minutes prior to the interview and not to climb stairs during the 15–30 minutes prior, and they were asked to rest for at least 5 minutes before measurements were taken. If either the systolic or diastolic readings differed by 5 mmHg from the first reading, measurements were repeated at an interval of 5–10 minutes. The process continued until two blood pressure values were obtained that did not vary by more than 5 mmHg in either the systolic or diastolic readings. The average of these two readings was used as the value for that particular participant [18]. The hypertension care cascade consists of five exclusive and independent categories that help to identify where the most significant loss of access to care occurs within the health system. These categories include people who are (1) unscreened (i.e. blood pressure ≥ 140/90 mmHg but never measured before and no reported diagnosis of hypertension); (2) screened but undiagnosed (i.e. blood pressure ≥ 140/90 mmHg and reporting having had blood pressure measured previously, but with no prior diagnosis of hypertension); (3) diagnosed but untreated (i.e. blood pressure ≥ 140/90 mmHg, previously diagnosed with hypertension, but no reported use of antihypertensive medication); (4) treated but uncontrolled (i.e. reported use of antihypertensive medication with blood pressure ≥ 140/90 mmHg); and (5) treated and controlled (i.e. reported use of antihypertensive medication with blood pressure < 140/90 mmHg) [11]. Using Yan et al.’s definition of unmet need as the total loss across all levels of the care cascade [9], the unmet need for hypertension care is the sum of all unscreened, undiagnosed, untreated and uncontrolled groups in the care cascade.

The ADL assessed were bathing, dressing, toilet use, transferring and self-control of continence, and feeding [19]. Responses to the queries about ADL were recorded as either independent or dependent. If the respondent answered even one of the questions about ADL component as “yes, dependent”, then they were categorized as “ADL dependent” and vice versa. Respondents were also asked about their history of diseases or comorbidities. These included stroke, heart disease, diabetes and hyperlipidaemia.

### Statistical analysis

SPSS version 27.0 for Windows (IBM Corporation, Armonk, NY, USA) was used for the statistical analysis. Descriptive statistics were calculated for several purposes: (1) to describe the prevalence of hypertension in the total sample; (2) to describe the proportion of hypertensive participants who were or were not ever screened, diagnosed or treated for hypertension, and who did or did not have normal blood pressure at the time of the survey; (3) to describe the percentage of hypertensive participants lost to care at each consecutive stage of the care cascade; and (4) to describe the characteristics of the sample with or without hypertension and with or without unmet need for care. Given that we had a binary outcome variable, logistic regression was chosen as the method for the inferential statistical analysis. Crude and adjusted odds ratios for the determinants of the unmet need for hypertension care were calculated using, respectively, simple logistic and multiple logistic regressions, with age group, gender, race, marital status, BMI, locality, smoking status, alcohol intake, medical history and ADL as covariates. Variables from the simple logistic regression with *P* < 0.05 were selected and forced into the multivariate model using the enter method. Sensitivity analysis was performed by repeating the analyses using hierarchical logistic regression with backward elimination of variables. All statistical tests were conducted at a 95% confidence interval (CI) using *P* < 0.05.

## Results

### Prevalence of hypertension

Of the total sample of 1204 participants, the majority were male (*n* = 691; 57%), of Malaysian ethnicity (*n* = 1002; 83%) and married (*n* = 802; 67%). Many were overweight (*n* = 501; 42%) and in the age group 60–64 years (*n* = 387; 32%) (Table 1). More than half of respondents (*n* = 637; 53%) were hypertensive. The hypertensive respondents displayed similar characteristics to the total sample. Most were in the 60–74 age range (*n* = 535; 84%), male (*n* = 352; 55%), Malaysian (*n* = 563; 88%) and married (*n* = 412; 65%). Among those with hypertension, many were overweight (*n* = 270; 42%), about one fifth were smokers (*n* = 114; 18%) and hardly any were alcohol drinkers (*n* = 11; 2%). For their medical history, 23 (4%) reported being previously diagnosed with stroke and 65 (10%) reported being diagnosed with cardiovascular disease; substantially more (*n* = 210; 33%) reported a history of diabetes mellitus or dyslipidaemia (*n* = 270; 42%) (Table 1).

**Table 1:** Characteristics of participants in the study of the hypertension care cascade, Malaysia

Participants (*N* = 1204)	Blood pressure				*P* value
	Normal (*n* = 567)		Hypertension (*n* = 637)		
	*n*	%	*n*	%	
Age (years)					
60–64	182	32	205	32	0.35
65–69	154	27	190	30	
70–74	125	22	140	22	
75–79	62	11	52	8	
80–84	37	7	35	6	
85–89	7	1	13	2	
90–94	0	0	2	0.3	
Gender					
Male	339	60	352	55	0.11
Female	228	40	285	45	
Race					
Malaysian	439	77	563	88	< 0.01*
Chinese	67	12	23	4	
Indian	58	10	50	8	
Other	3	1	1	0.2	
Marital status					
Married	390	69	412	65	0.13
Other	177	31	225	35	
Body mass index					
Underweight	22	4	17	3	0.07
Normal	202	36	193	30	
Overweight	231	41	270	42	
Class I obesity	74	13	117	18	
Class II obesity	31	6	30	5	
Class III obesity	7	1	10	2	
Locality					
Rural	265	47	337	53	0.03*
Urban	302	53	300	47	
Smoking					
Yes	101	18	114	18	0.97
No	466	82	523	82	
Alcohol intake					
Yes	31	6	11	2	< 0.01*
No	536	95	626	98	
Medical history					
Stroke					
Yes	25	4	23	4	0.48
No	542	96	614	96	
Cardiovascular disease					
Yes	74	13	65	10	0.12
No	493	87	572	90	
Diabetes mellitus					
Yes	224	40	210	33	0.02*
No	343	61	427	67	
Dyslipidaemia					
Yes	300	53	270	42	< 0.01*
No	267	47	367	58	
Activities of daily living					
Independent	34	6	59	9	0.03*
Dependent	533	94	578	91	

*Significant at *P* < 0.05

### Hypertension care cascade

**Fig. 1:**
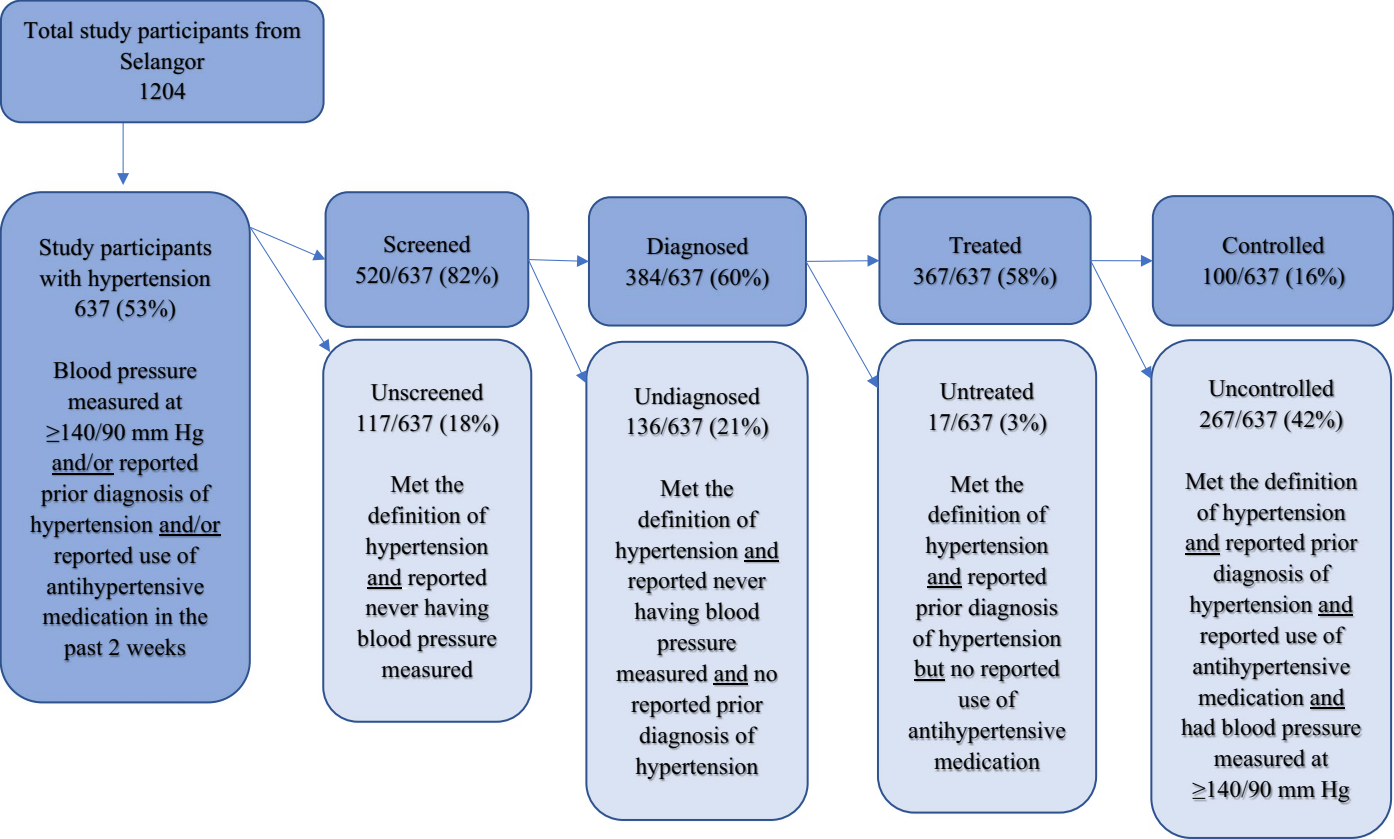
Classification of study participants across the hypertension care cascade

Participants were assigned to a specific group in the care cascade based on their blood pressure measurements and survey responses (Fig. 1). Among the 637 (53%) participants who met the definition of hypertension, 18% (*n* = 117) had never been screened, 21% (*n* = 136) had been screened but were undiagnosed, 3% (*n* = 17) had been screened and diagnosed but had not been treated with antihypertensive medication, 42% (*n* = 267) were being treated with antihypertensive medication but still had high blood pressure, and 16% (*n* = 100) had hypertension controlled with medication. Thus, effective coverage of hypertension treatment that successfully controlled blood pressure for this group of older hypertensive persons was only 16%.

**Fig. 2:**
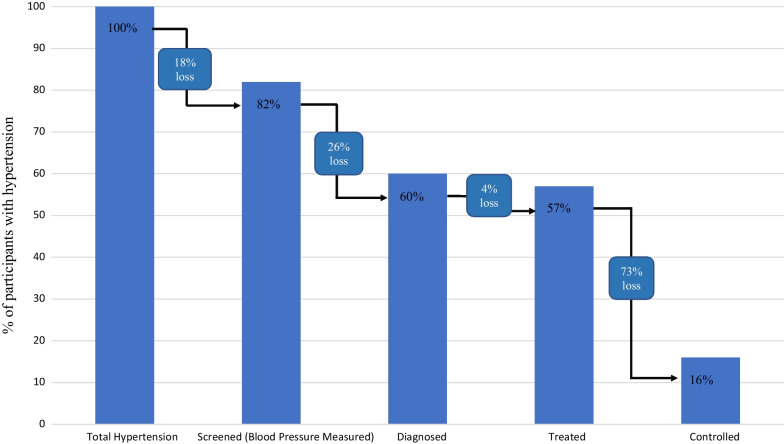
Loss to care at each stage of the hypertension care cascade. Of those with hypertension, 82% had been screened for hypertension, representing an 18% loss to screening. Of those who had ever had their blood pressure measured, 74% received a diagnosis of high blood pressure, representing a 26% loss to diagnosis. Of those who had been diagnosed, 96% were being treated with antihypertensive medication, a 4% loss to treatment. Of those who had taken blood pressure medication in the past 2 weeks, 27% had controlled blood pressure (< 140/90 mmHg), a 73% loss to effective treatment

The loss of care across the hypertension care cascade is displayed in Fig. 2. The first stage in the cascade is to be screened for hypertension through blood pressure measurement. Among those with hypertension, 82% (520/637) reported that they had ever had their blood pressure measured, indicating an 18% (117/637) loss to screening. Among those who reported ever being screened, 74% (384/520) said that they had been diagnosed with hypertension by a health professional. Among those who reported a prior diagnosis of hypertension, 96% (367/384) also reported being prescribed blood pressure medication. Finally, among those treated for hypertension, only 27% (100/367) had controlled blood pressure (< 140/90 mmHg). This is where the greatest loss of care, or loss to effective treatment, occurs: a loss of 73%.

### Prevalence of unmet needs by stage of the hypertension care cascade

Table 2 shows that 537 (84%) of the 637 hypertensive participants had unmet needs for hypertension care. Four subgroups were defined as having specific unmet needs for care: those who were unscreened (117/637; 18%), undiagnosed (136/637; 21%) or untreated (17/637; 3%), and those who were treated but whose hypertension was uncontrolled (267/637; 42%). The demographic characteristics of the subgroups with unmet needs were similar to one another and to the overall population characteristics, which means they were mostly aged 60–74 years, male, Malaysian and married. They were also like one another and to the overall study sample in that hardly any were alcohol drinkers, only a few had a history of stroke or cardiovascular disease, and most were dependent on others to perform ADL.

**Table 2:** Characteristics of the study population with unmet needs for hypertension care, Malaysia

Characteristics (*N* = 537)	Hypertension							
	Unscreened (*n* = 117)		Undiagnosed (*n* = 136)		Untreated (*n* = 17)		Uncontrolled (*n* = 267)	
	*n*	%	*n*	%	*n*	%	*n*	%
Age (years)								
60–64	40	34	49	36	6	35	77	29
65–69	31	27	49	36	5	29	77	29
70–74	30	26	18	13	6	35	66	25
75–79	9	8	12	9	0	0	20	8
80–84	6	5	4	3	0	0	19	7
85–89	1	1	3	2	0	0	7	3
90–94	0	0	1	1	0	0	1	0.4
Gender								
Male	68	58	79	58	10	59	140	52
Female	49	41	57	42	7	41	127	48
Race								
Malaysian	101	86	121	89	15	88	231	87
Chinese	6	5	8	6	0	0	9	3
Indian	10	9	7	5	2	12	27	10
Other	0	0	0	0	0	0	0	0
Marital status								
Married	74	63	99	73	11	65	168	63
Other	43	37	37	27	6	35	99	37
Body mass index								
Underweight	3	3	7	5	1	6	4	2
Normal	51	44	57	42	5	29	53	20
Overweight	38	33	46	34	8	47	133	50
Class I obesity	19	16	19	14	2	12	57	21
Class II obesity	4	3	6	4	0	0	18	7
Class III obesity	2	2	1	1	1	6	2	1
Locality								
Rural	74	63	62	46	12	71	132	49
Urban	43	37	74	54	5	29	135	51
Smoking								
Yes	30	26	98	72	3	18	33	12
No	87	74	38	28	14	82	234	88
Alcohol intake								
Yes	4	3	2	2	0	0	5	2
No	113	97	134	99	17	100	262	98
Medical history								
Stroke								
Yes	1	1	2	2	0	0	12	5
No	116	99	134	99	17	100	255	96
Cardiovascular disease								
Yes	4	3	9	7	1	6	42	16
No	113	97	127	93	16	94	225	84
Diabetes mellitus								
Yes	11	9	20	15	3	18	132	49
No	106	91	116	85	14	82	135	51
Dyslipidaemia								
Yes	12	10	35	26	3	18	171	64
No	105	90	101	74	14	82	96	36
Activities of daily living								
Independent	12	10	4	3	1	6	32	12
Dependent	105	90	132	97	16	94	235	88

### Determinants of unmet need for care among older people with hypertension

Of all the covariates considered, when using simple logistic regression only four factors were associated with a significantly higher odds of having any unmet need for hypertension care among the older population in Selangor, namely, class III obesity, being a nonsmoker and having a history of stroke or diabetes (Table 3). Multivariate analysis (Table 4) identified three significant factors associated with an unmet need for hypertension care: being a nonsmoker and having a history of stroke or diabetes mellitus. Smokers have a 50% reduced risk of having an unmet need for hypertension care compared with nonsmokers after adjusting for past diagnosis of stroke or diabetes (adjusted odds ratio = 0.5; 95% CI: 0.3–0.9). Older adults who had a history of stroke or diabetes mellitus had higher odds of having an unmet need for hypertension care compared with those without such a history, when adjusting for other factors: 2.8 (95% CI: 1.1–6.9) for history of stroke and 1.6 (95% CI: 1.0–2.5) for history of diabetes mellitus. These results were not significantly changed when the analysis was repeated using hierarchical logistic regression instead of forced variable entry, in which demographic variables were entered in the first block, followed by health risk factors in the second block and medical history and function in the third block, with backward elimination of variables from the model.

**Table 3:** Univariate analysis of predictors of total unmet need for hypertension care among participants with hypertension, Malaysia

Characteristics (*N* = 637)	Predictors of total unmet need		
	Odds ratio	95% confidence interval	*P* value
Age (years)			
60–64	Reference		
65–69	0.9	0.5–1.6	0.71
70–74	0.9	0.5–1.6	0.65
75–79	1.4	0.7–2.9	0.29
80–84	1.1	0.4–2.8	0.88
85–89	0.9	0.2–4.4	0.95
90–94	0.0	NA	1.00
Gender			
Male	Reference		
Female	1.0	0.7 to 1.6	0.95
Race			
Malaysian	Reference		
Chinese	0.0	0.0 to 0.4	0.99
Indian	0.43	0.2 to 1.2	0.11
Other	0.26	0.0 to 2.5	0.24
Marital status			
Married	Reference		
Other	1.3	0.8 to 1.9	0.3
Body mass index			
Normal	Reference		
Class I obesity	1.3	0.6 to 2.4	0.46
Class II obesity	0.4	0.1 to 1.9	0.28
Class III obesity	4.9	1.1 to 15.5	0.04*
Overweight	1.2	0.7 to 2.1	0.43
Underweight	0.8	0.2 to 3.8	0.79
Locality			
Urban	Reference		
Rural	1.2	0.8 to 1.9	0.37
Smoking			
No	Reference		
Yes	0.5	0.2 to 0.9	0.03*
Medical history			
Stroke			
No	Reference		
Yes	3.0	1.2 to 7.3	0.01*
Cardiovascular disease			
No	Reference		
Yes	0.8	0.4–1.8	0.70
Diabetes mellitus			
No	Reference		
Yes	1.8	1.1–2.7	0.01*
Dyslipidaemia			
No	Reference		
Yes	1.4	0.9–2.1	0.15
Activities of daily living			
Independent	Reference		
Dependent	0.9	0.4–1.8	0.80

*NA* not applicable

*Significant at *P* < 0.05

**Table 4:** Multivariate predictors of unmet needs for hypertension care among participants with hypertension, Malaysia

Characteristics (*N* = 537)	Predictors of unmet needs		
	Adjusted odds ratio^a^	95% confidence interval	*P* value
Body mass index			
Normal	Reference		
Class I obesity	1.1	0.6–2.1	0.81
Class II obesity	0.4	0.1–1.7	0.20
Class III obesity	2.9	0.7–11.4	0.13
Overweight	1.1	0.7–1.9	0.67
Underweight	0.8	0.2–4.2	0.89
Smoking			
No	Reference		
Yes	0.5	0.3–0.9	0.05*
Medical history			
Stroke			
No	Reference		
Yes	2.8	1.1–6.9	0.03*
Diabetes mellitus			
No	Reference		
Yes	1.6	1.0–2.5	0.05*

*Significant at *P* < 0.05

^a^Enter method used for multiple logistic regression

We also analysed the possible determinants of loss of care at each stage of the care cascade using the same set of covariates and simple and multiple logistic regression analyses. Among people with hypertension (*n* = 637), those with a prior diagnosis of diabetes (adjusted odds ratio = 3.6; 95% CI: 1.8–7.2) or dyslipidaemia (adjusted odds ratio = 5.5; 95% CI: 2.8–10.6) were more likely to be unscreened. Among people with hypertension who had been screened previously (*n* = 520) those who were more likely to be undiagnosed were aged 70–74 years (adjusted odds ratio = 2.8; 95% CI: 1.4–5.5, with 60–64 year olds as the reference group), had a BMI classification of underweight (adjusted odds ratio = 2.2; 95% CI: 1.1–4.4, with normal weight as the reference group) or class II obesity (adjusted odds ratio = 2.6; 95% CI: 1.5–4.5, with normal weight as the reference group), and had a history of diabetes (adjusted odds ratio = 5.5; 95% CI: 2.8–10.6) or dyslipidaemia (adjusted odds ratio = 5.5; 95% CI: 2.8–10.6). Conversely, those who were less likely to be undiagnosed were smokers (adjusted odds ratio = 5.5; 95% CI: 2.8–10.6) and those who were ADL-dependent (adjusted odds ratio = 5.5; 95% CI: 2.8–10.6). Among the people with hypertension who had been diagnosed previously (*n* = 384), having a history of dyslipidaemia (adjusted odds ratio = 5.7; 95% CI: 1.6–20.4) was the only statistically significant predictor of being untreated. Lastly, of those who had taken antihypertensives in the preceding 2 weeks (*n* = 367), the only statistically significant predictor of having uncontrolled blood pressure was not having a history of dyslipidaemia (simple odds ratio = 0.5; 95% CI: 0.3–0.9). It should be noted that the sample size reduces in these analyses as we progress through the care cascade because the denominator changes.

## Discussion

Several critical findings were revealed in this study. The prevalence of hypertension is high among the population aged ≥ 60 years in Selangor, affecting more than half (53%) of the study’s participants. This is lower than the prevalence of 74.1% reported in 2011 for people aged 60–69 years in Malaysia [6] or 75.9% reported in 2015 for the same age group in the neighbouring country of Brunei Darussalam [20]. Nonetheless, it is higher than previously reported rates of hypertension among adult populations in Selangor (12.7% among adults aged ≥ 18 years in 2019) [7], in Malaysia (43.5% among adults aged ≥ 30 years in 2011) [6] and globally (32% among women and 34% among men aged 30–79 years in 2019) [21], and it is still a cause for concern.

Moreover, this study reports one of the first estimates of the extent of unmet needs for hypertension care and its possible predictors among the older population in Selangor. There is a significant unmet need for hypertension care in the study sample: 84% of those with hypertension have unmet needs for care at some stage of the care cascade. The high level of unmet need for hypertension care was mainly attributed to the loss of care at the stage of control, or effective treatment, which contributed 50% to the total unmet need.

The typical patient with hypertension is initially asymptomatic. Additionally, older people commonly view hypertension as a normal part of ageing. These tendencies make it challenging to motivate older people to seek care or to engage in self-care to control the disease [22]. This is also why it is challenging to achieve a treatment goal of blood pressure < 140/90 mmHg in older patients [23]. The levels of screening (82%), diagnosis or awareness (60%), treatment (58%) and control (16%) among older hypertensive people in this study compare favourably to reported figures for adults aged ≥ 30 years in Malaysia in 2006 (31.5% of men and 40.0% of women were diagnosed or aware; 7.7% of men and 8.7% of women had blood pressure controlled by treatment) [6] and those reported in another study of adults aged ≥ 20 years in Malaysia in 2011 (39.0% were diagnosed or aware; 35.7% were being treated; 9.6% had blood pressure controlled by treatment) [24]. The coverage rates in this study are similar to those reported for hypertensive adults aged ≥ 18 years in Brunei Darussalam in 2015 [20] (67% were diagnosed or aware; 52% were being treated; and 27% had controlled blood pressure), but there were slightly worse treatment outcomes. The coverage of hypertension care found among hypertensive older people in this study is also better than the average levels of coverage reported among hypertensive populations aged ≥ 15 years in 44 low- and middle-income countries, based on the latest available nationally representative data since 2005 (73.6% were screened; 39.2% were diagnosed; 29.9% were being treated; and 10.3% had controlled blood pressure) [25], and they are closer to the global averages reported for adults aged 30–79 when using data from 200 countries and territories of all income levels in 2019 (59% of women and 49% of men were diagnosed; 47% of women and 38% of men were being treated; and 23% of women and 18% of men had controlled blood pressure) [21].

The greatest loss of care in this sample occurred at the stage of control, or effective treatment—a 73% loss—with the loss of care at other stages being significantly less (4–26%). The combined loss of screening and diagnosis is about 40%, far less than the 73% loss at the control stage. This contrasts with the results of a global study showing that loss of hypertension care tends to be greatest at the diagnosis stage (including those who are screened and unscreened) and the control stage, with around 50% loss at each stage [21]. The results from the present study also differ from the findings of the few studies on the hypertension care cascade conducted in low- and middle-income countries. A study of only low- and middle-income countries found that loss to care was highest at the diagnosis and control stages, with 61% loss and 66% loss, respectively [25]. Studies from Nigeria [26], Sierra Leone [27] and South Africa [11] also show that the greatest loss to care happens at the screening and diagnosis stages, and although the loss to care at the control stage was also high, it did not reach the level observed in the present study. Thus, the high level of loss to care at the control stage is a relatively unique characteristic of this study. This finding is consistent with another study in Malaysia on adult hypertension that showed the same level of loss to care at the control stage (73%), which implies that this pattern may apply to Malaysia more broadly and not just to Selangor [24].

These results indicate that assessments of service coverage for hypertension that rely only on measures of diagnosis or treatment coverage will severely underestimate the unmet need for effective treatment and blood pressure control in this context, where loss to care at the control stage is high.

The reasons for ineffective treatment could be explained by several factors, such as nonadherence to treatment, using nonoptimal doses of antihypertensive medication, experiencing adverse events associated with medications, polypharmacy or the presence of hypertension-related comorbidities and complications. The high prevalence of nonadherence to antihypertensive medication has been shown in a systematic review, which found it to be particularly prevalent among patients with uncontrolled blood pressure (83.7%) [28].

Our analysis showed that the significant determinants for having any unmet need in the care cascade were being a nonsmoker and having a history of stroke or diabetes mellitus. Having a history of dyslipidaemia was a consistent predictor of loss to screening, loss to diagnosis and loss to treatment, but once people began treatment, those with a history of dyslipidaemia were more likely to have their blood pressure controlled.

Compared with nonsmokers, older people in this study who smoke have a 50% lower risk of having unmet needs for hypertension care. Smoking is a risk factor for developing hypertension, as supported by a study in Japan that concluded that smoking exposure was associated with the prevalence of hypertension [29]. However, a survey of high-risk smokers showed that older smokers are aware of the health risks posed by smoking and are interested in health screenings [30]. It has also been demonstrated that doctors perceive older men with high-risk behaviours such as smoking to be more receptive to health screening and more likely to discuss health screening [31]. Therefore, the evidence suggests that older people with high-risk behaviours such as smoking may have a greater tendency to seek treatment or be prioritized for health screenings by healthcare providers, thus reducing their risk of having unmet needs for hypertension care.

Hypertensive patients with diabetes mellitus have variability in their blood pressure, and their hypertension is more resistant to treatment, leading to uncontrolled hypertension [32]. Additionally, diabetes mellitus is one of the leading causes of end-stage renal disease, and hypertension is also a risk factor for end-stage renal disease. Patients with end-stage renal disease need frequent dialysis, which may mean it is more likely that they will miss appointments related to treatment for their hypertension [33]. Diabetes mellitus also causes diabetic retinopathy, and hypertension accelerates the development of diabetic retinopathy, which may make it difficult for patients to take their medication correctly and follow up on hypertension appointments due to their impaired vision [34]. All the studies mentioned indicate that hypertensive patients with underlying diabetes mellitus have unmet needs for hypertension care due to complications caused by both diseases.

Stroke is another predictor of having an unmet need for hypertension care in this study. Most stroke patients have underlying hypertension, which is the most important modifiable risk factor for stroke [35]. A study showed that nonadherence to secondary preventive medications after stroke is relatively common [36]. Another study showed that stroke patients have high unmet needs for healthcare [37]. A further study in the United States found that patients with stroke face problems from nonresponsive health systems due to their changing needs and a lack of regular reassessment, thus leading to unmet needs for hypertension care [38]. Stroke survivors who require assistance with ADL reported having more unmet healthcare needs than stroke patients who are ADL-independent [39]. So, it may also be possible that stroke patients with underlying hypertension and disability are more likely to have unmet needs for healthcare and hypertension care due to limitations in ADL that make it harder for them to attend clinic appointments and make them prone to nonadherence to medication and treatment. As this is a cross-sectional study, a causal association cannot be established, nor can its direction. Thus, it is possible that those with a history of stroke are more likely to have unmet needs for hypertension care, as suggested here, but it is also possible that those with unmet needs for hypertension care are more likely to suffer stroke, or both.

Regardless of the causal nature of the associations, these findings suggest that certain groups of older people should be given particular attention to ensure they receive proper care to prevent hypertension incidence, recurrent hypertension or complications from hypertension comorbidity. This includes people with comorbidities or relevant medical history, such as people with diabetes or stroke patients; people who may otherwise seem to be at low risk, including being a nonsmoker or non-alcohol drinker; and possibly people who have limitations in ADL. When sample sizes are sufficiently large, an analysis of the predictors of unmet need at each stage of the care cascade can be helpful to determine those who are most at risk of having an unmet need for each type of care. As our analysis for a history of dyslipidaemia showed, the same factor could have a barrier effect at some stages of care (e.g. screening, diagnosis, and treatment) while having a facilitator effect at other stages (e.g. effective treatment and blood pressure control). This would also have implications for health system strengthening that aims to close service gaps since the actors differ at each stage of care, for example, between screening and treatment.

This and other studies have shown that critical gaps in hypertension management identified by using the care cascade occur at the control, screening and diagnosis stages. However, there may be ways to bridge these gaps. Older patients with chronic illnesses often have a lower perception of the severity of their disease and a negative attitude towards treatment [40], which leads to medication nonadherence. To resolve this, counselling and health education can be offered while patients are waiting for their healthcare appointments, and these services can emphasize the consequences of nonadherence and the importance of taking medications on time. The detrimental effects of mixing modern with traditional medicine, which is a fairly common practice in Malaysia, should also be stressed. Additionally, it is critical to improve doctor–patient relationships so that patients are more comfortable discussing treatment-related issues [41].

There are also gaps between guidelines and actual clinical practices, which lead to a lower prevalence of diagnosis and suboptimal care among hypertension patients in Malaysia [42]. Prompt re-evaluation of the current approaches and strategies used to diagnose and treat hypertension is vital to improving the quality of care and outcomes among hypertension patients. The clinical guidelines used to define and detect hypertension may also make a difference. For example, the American College of Cardiology/American Heart Association guidelines from 2020 define hypertension as systolic pressure ≥ 130 mmHg and/or diastolic pressure ≥ 80 mmHg (stage 1 hypertension) [43], which are lower than the thresholds used in the 2018 Malaysia guideline on the management of hypertension [17]. As treatment for chronic diseases evolves, it is crucial to review the situation in each country and update guidelines for clinical management. Having a lower threshold for the definition of hypertension can facilitate implementation of interventions at an earlier stage of disease and may improve the ability to manage the disease and to prevent complications.

In Malaysia, healthcare is provided through collaboration between the public and private sectors. Screening, diagnosis, and treatment are carried out mainly by primary care providers [44]. The public sector provides direct care to the rural population through health and community clinics, while private primary care providers complement the public sector by offering services in urban areas for those who can afford them [45]. Government health clinics screen patients opportunistically, which explains why there is higher coverage of hypertension diagnosis among women (i.e. screening is done as they attend for maternal and child health services) and among persons aged ≥ 60 years (i.e. among those attending for chronic disease care and minor ailments) [46]. For countries like Malaysia that need to adapt and strengthen their primary care services for managing hypertension and cardiovascular disease, the HEARTS technical package may be useful. Developed under the auspices of the Global Hearts Initiative, led by WHO and the United States Centers for Disease Control and Prevention, the HEARTS technical package provides solutions to improve the control of hypertension at the primary care level that have been shown to be effective, affordable, and scalable in a wide range of low- and middle-income countries [47].

Finally, the limitations of this study should be noted. First, recall bias may have affected the reporting of being screened, diagnosed, or treated for hypertension in this cross-sectional study. Collecting data in real time and using longitudinal follow-up for hypertensive patients may provide a more accurate assessment of where the loss occurs in the care cascade. However, all teams conducting the interviews had been trained to minimize potential recall bias. The same survey questions are commonly used in other health surveys and in analyses of service coverage based on the hypertension care cascade, as reported in other studies cited in this paper, which facilitates comparability. Second, this study employed two blood pressure measurements during a single visit to diagnose hypertension instead of the standard in the 2018 Malaysian clinical practice guidelines, which recommends measuring blood pressure on at least two separate occasions for diagnostic purposes [17]. However, several other studies and national surveys have also used a method similar to that in the current study due to the difficulties in measuring blood pressure on two separate visits in extensive population surveys [48]. Third, the ethnic population in this study does not reflect Selangor’s ethnic population, which comprises 55% Malaysian, 24.1% Chinese, 11.2% Indian and 0.8% other groups [49]. Malaysians were overrepresented, while the Chinese and Indian populations were underrepresented, as were those of other ethnic groups. Lastly, as this is a cross-sectional study, the causal directions of the associations identified in this study are unclear. Nonetheless, the care cascade is a useful framework that can be applied to cross-sectional data to obtain insight into unmet needs for hypertension care and the possible bottlenecks or gaps in service delivery.

## Conclusions

There is likely a significant unmet need for hypertension care, from screening to effective treatment, among older individuals in the state of Selangor, Malaysia. Older people who have comorbidities, who have behavioural risk factors that put them at low risk and who have limitations in ADL may be at higher risk of having an unmet need. Although several national policies to improve hypertension screening and care have been implemented, our study showed that significant gaps may remain. Research using the care cascade as the analytical framework is particularly useful for understanding where people with hypertension are lost by the healthcare system. In this study, the most significant loss to care occurred at the control stage, followed by the screening and diagnosis stages. As Malaysia will transition to become an ageing nation by 2030, and this will be accompanied by a shift in disease burden from communicable diseases to noncommunicable diseases, the healthcare system must strengthen its capacity to provide quality care for hypertension and other chronic conditions to reduce unmet needs for healthcare among older people. Research can play a critical role in identifying where capacity needs to be strengthened. This strengthening could include increasing the number of primary healthcare clinics and staff, as well as other health providers, such as community pharmacists, who could be trained and equipped with necessary supplies to manage noncommunicable diseases such as diabetes and hypertension. Such strengthening would also require better integration and coordination across different care levels and settings to provide person-centred care. Future research should include socioeconomic status and access to healthcare services and hospitals as other possible determinants for unmet needs of chronic disease care, and could use prospective cohort studies to evaluate the progression of hypertension complications, patients’ utilization and receipt of care, and the associated healthcare costs among individuals with unmet needs for hypertension care.

## Data Availability

The data sets used and analysed in this study are available from the corresponding author upon reasonable request.

## Abbreviations

CI: Confidence interval
BMI: Body mass index
ADL: Activities of daily living
